# Diel activity patterns of two distinct populations of *Aedes aegypti* in Miami, FL and Brownsville, TX

**DOI:** 10.1038/s41598-022-06586-w

**Published:** 2022-03-29

**Authors:** John-Paul Mutebi, André Barretto Bruno Wilke, Erik Ostrum, Chalmers Vasquez, Gabriel Cardenas, Augusto Carvajal, Maday Moreno, William D. Petrie, Arturo Rodriguez, Henry Presas, Jesus Rodriguez, Fred Barnes, Gabriel L. Hamer, Jose G. Juarez, Ester Carbajal, Christopher J. Vitek, Xochitl Estrada, Thalia Rios, Jeremy Marshall, John C. Beier

**Affiliations:** 1 Arboviral Diseases Branch (ADB), Division of Vector-Borne Diseases (DVBD), Centers for Disease Control and Prevention (CDC), 3156 Rampart Road, Fort Collins, CO 80521 USA; 2 Miami-Dade County Mosquito Control Division, Miami, FL USA; 3 Department of Public Health Sciences, Miller School of Medicine, University of Miami, 1120 Northwest 14th Street, Miami, FL 33136 USA; 4 City of Brownsville, Public Health Department, 1034 E. Levee St., Brownsville, TX 78521 USA; 5 Department of Entomology, Texas A&M University, College Station, TX USA; 6 Center for Vector-Borne Diseases, The University of Texas Rio Grande Valley, Edinburg, TX 78539 USA

**Keywords:** Ecology, Zoology

## Abstract

The diel biting activity of *Aedes* (*Stegomyia*) *aegypti* (L) populations was extensively investigated in the early 1900s to gain more information on the biology of *Ae. aegypti*, and this information was used to devise effective approaches to controlling populations of this species and protect the human population from widespread arbovirus outbreaks. However, few contemporary studies are available regarding the diel activity patterns of *Ae. aegypti*. To assess the diel activity patterns of *Ae. aegypti* in southern Florida and Texas, we conducted 96-h uninterrupted mosquito collections once each month from May through November 2019 in Miami, Florida, and Brownsville, Texas, using BG-Sentinel 2 Traps. The overall diel activity pattern in both cities was bimodal with morning and evening peak activity between 7:00 and 8:00 and between 19:00 and 20:00. There were significant daily, monthly, seasonal, and site-specific differences in activity patterns, but these differences did not affect the overall peak activity times. These differences suggest daily, monthly, seasonal, and site-specific variations in human exposure to *Ae. aegypti*. Our observations can be used in planning and executing *Ae. aegypti* vector control activities in southern Florida and southern Texas, specifically those targeting the adult mosquito populations.

## Introduction

The diel biting activity of *Aedes* (*Stegomyia*) *aegypti* (L) populations was extensively investigated in the early part of the twentieth century. The primary aim was to gain more information on the biology of *Ae. aegypti* and use this information to devise effective approaches to controlling populations of this species and protect the human population from widespread urban yellow fever (YF) outbreaks. The bulk of the investigations were conducted in the tropical regions of South and Central America, and East and West Africa. Most of the early studies and the observations were summarized in the monograph by Christophers^1^. The general observations were that *Ae. aegypti* activity was diurnal and it was widely referred to as a daytime biting mosquito^1^. However, there were several studies that detected substantial nighttime biting activity in some populations of *Ae. aegypti*. For example, Lumsden^2^ reported that approximately 34% of the *Ae. aegypti* population in the Southern Province of Tanganyika, in East Africa, were biting at night. In addition, distinct after-dark biting activity peaks were observed indoors but not outdoors^2^ suggesting behavioral adaptations of *Ae. aegypti* populations to local environmental conditions. Taken together these observations suggested variations in diel host-seeking activity among geographic populations of *Ae. aegypti*.

Later studies in Tanzania reported that the diel biting activity had two peaks, one in the morning and one in the evening^3^. Similarly, a study in Trinidad detected morning and evening peaks however, this study also detected a third peak at 11:00^4^. This suggested a trimodal pattern of *Ae. aegypti* diel biting activity in Trinidad. The study by Diarrassouba and Dossou-Yovo^5^ pointed out that although the biting activity of *Ae. aegypti* is diurnal in East Africa, the peak biting activity was usually at sunset in West Africa. Furthermore, they reported an unusual diel biting activity in the savannah zone of Côte d'Ivoire in the dry season where *Ae. aegypti* showed an atypical biting activity rhythm in the dry season, and biting activity was throughout the night and peaking at midnight^5^. A recent study by Ortega‑López et al.^6^ used BG‑Sentinel traps and Mosquito electrocuting traps to study host-seeking activity of *Ae. aegypti* in Quinindé, Ecuador. They found that female *Ae. aegypti* had bimodal patterns of host-seeking with a peak in the early morning and another peak in the late afternoon.

There have been very few studies in the contiguous United States (CONUS), and to our knowledge, the only published study from the CONUS is that by Smith et al.^7^. This study was conducted in Saint Augustine, Florida and determined that the evening activity was the most significant, with peak activity occurring between 17:00 and 19:00, followed by a period of substantial host-seeking activity between 19:00 and 21:00. They also detected a minor peak between 7:00 and 9:00. They noted that there was a gradual increase in activity leading up to the peaks and a gradual decrease in activity from the peak hours.

Following the Zika outbreak in the United States in 2016, there was a realization that not much was known about the distribution and the diel host-seeking activity patterns of *Ae. aegypti* populations in the CONUS. In 2019, we initiated a study with the aim to increase our understanding of the diel host-seeking activity patterns of *Ae. aegypti* in two cities in the southeastern United States. An understanding of the host-seeking diel patterns of mosquitoes can greatly improve the effectiveness of mosquito control by guiding adulticiding and other integrated mosquito control activities.

## Results

### Mosquito species captured

A total of 26 mosquito species were collected, 17 species in Brownsville, TX and 18 in Miami, FL (Table 1). The most frequently captured species at both sites was *Ae. aegypti*, 61.33% (13,033/21,252) of the collection in Brownville, TX, and 48.74% (6461/13,257) of the collections in Miami, FL. This is not surprising because the BG-Sentinel 2 traps we used in this study were specifically designed to capture *Ae. aegypti*^8,9^. The second and third most captured species captured in Brownsville, TX, were *Culex* (*Culex*) *quinquefasciatus* Say, 24.55% (5217/21,252), and *Aedes* (*Aedimorphus*) *vexans* (Meigen), 5.39% (1145/21,252), respectively. In Miami, FL, *Wyeomyia* (*Wyeomyia*) *vanduzeei* Dyar & Knab 19.94% (2644/13,257) and *Aedes* (*Ochlerotatus*) *taeniorhynchus* (Wiedemann) 19.93% (2642/13,257), were the second and third most captured species respectively (Table 1). Of the 26 species captured, only 9, (34.62%) were captured in both Brownsville and Miami (Table 1). Six species were unique to the Brownsville collections, and 9 species were captured only in Miami (Table 1).

**Table 1:** Mosquito species captured by using BG-Sentinel 2 traps during *Aedes aegypti* activity pattern studies in Brownsville, Texas and Miami, Florida in 2019.

Species	Brownsville			Miami		
	Females	Males	Total	Females	Males	Total
*Aedes aegypti*	7024	6009	13,033	4444	2017	6461
*Aedes albopictus*	424		424	1		1
*Aedes infirmatus*	395		395	2		2
*Aedes sollicitans*	58		58			
*Aedes taeniorhynchus*	420		420	2580	62	2642
*Aedes thelcter*	12		12			
*Aedes tortilis*				629		629
*Aedes triseriatus*				6	1	7
*Aedes vexans*	1145		1145			
*Aedes zoosophus*	1		1			
*Anopheles atropos*				1		1
*Anopheles crucians*				12		12
*Anopheles pseudopunctipennis*	27		27			
*Anopheles quadrimaculatus*				18		18
*Culex biscaynensis*				14	16	30
*Culex coronator*	332		332	194	23	217
*Culex erraticus*	9		9	8		8
*Culex nigripalpus*	113		113	91	6	97
*Culex quinquefasciatus*	4473	744	5217	237	175	412
*Culex* spps				1	3	4
*Deinocerites cancer*				16		16
*Psorophora ciliata*	1		1			
*Psorophora columbiae*	33		33	42	3	45
*Psorophora cyanescens*	28		28			
*Toxorhynchites rutilus septentrionalis*	4		4			
*Wyeomyia mitchellii*				11		11
*Wyeomyia vanduzeei*				2639	5	2644

### Diel activity patterns

**Figure 1:**
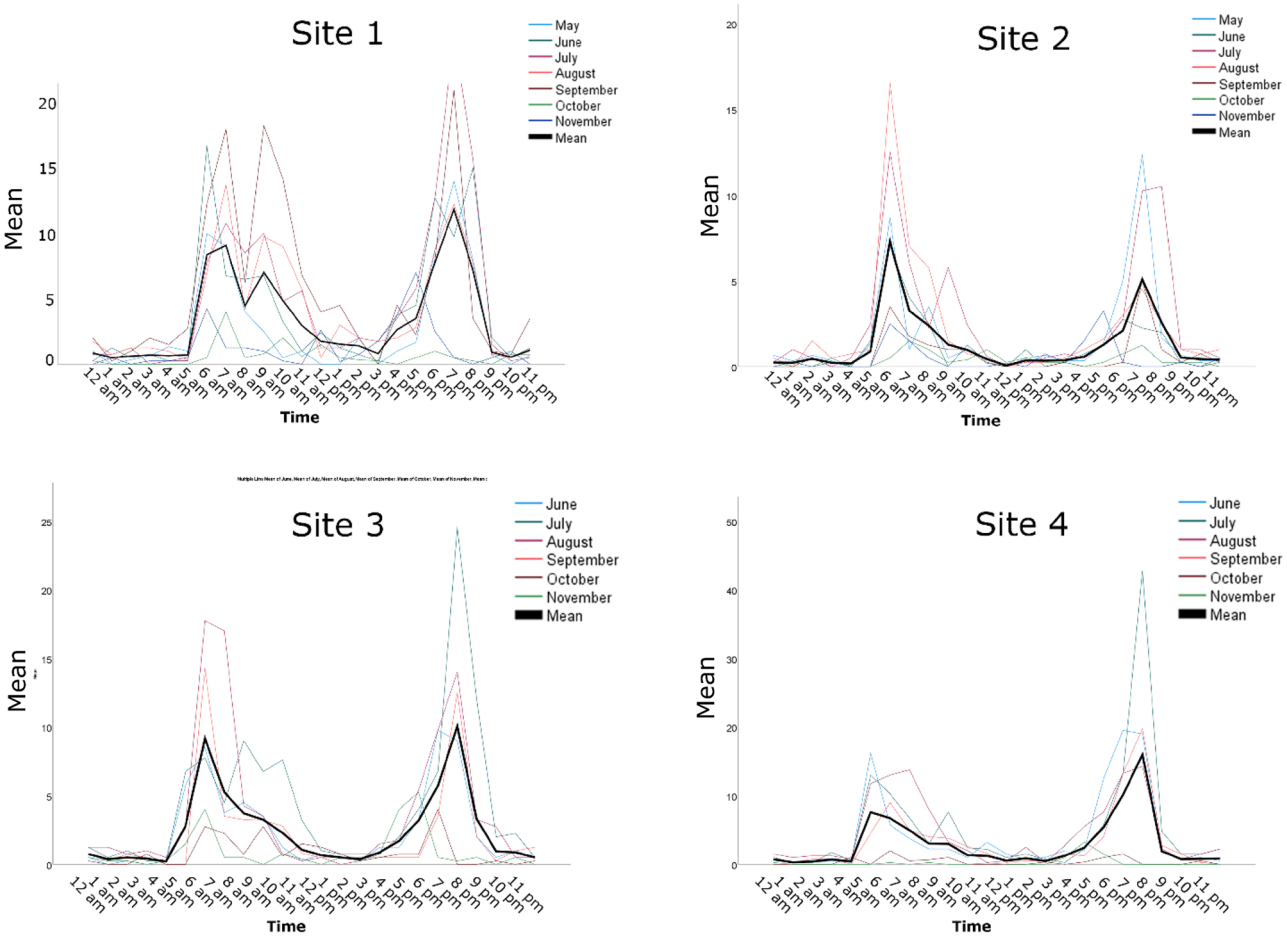
Diel activity patterns for *Ae. aegypti* populations at each of the 4 sampling sites in Miami, Florida. “Mean” on the graphs represent the mean number of female *Ae. aegypti* mosquitoes captured per trap/hour. The thick black line is the mean number of *Ae. aegypti* female mosquitoes captured per trap/hour throughout the study period from May to November 2019.

**Figure 2:**
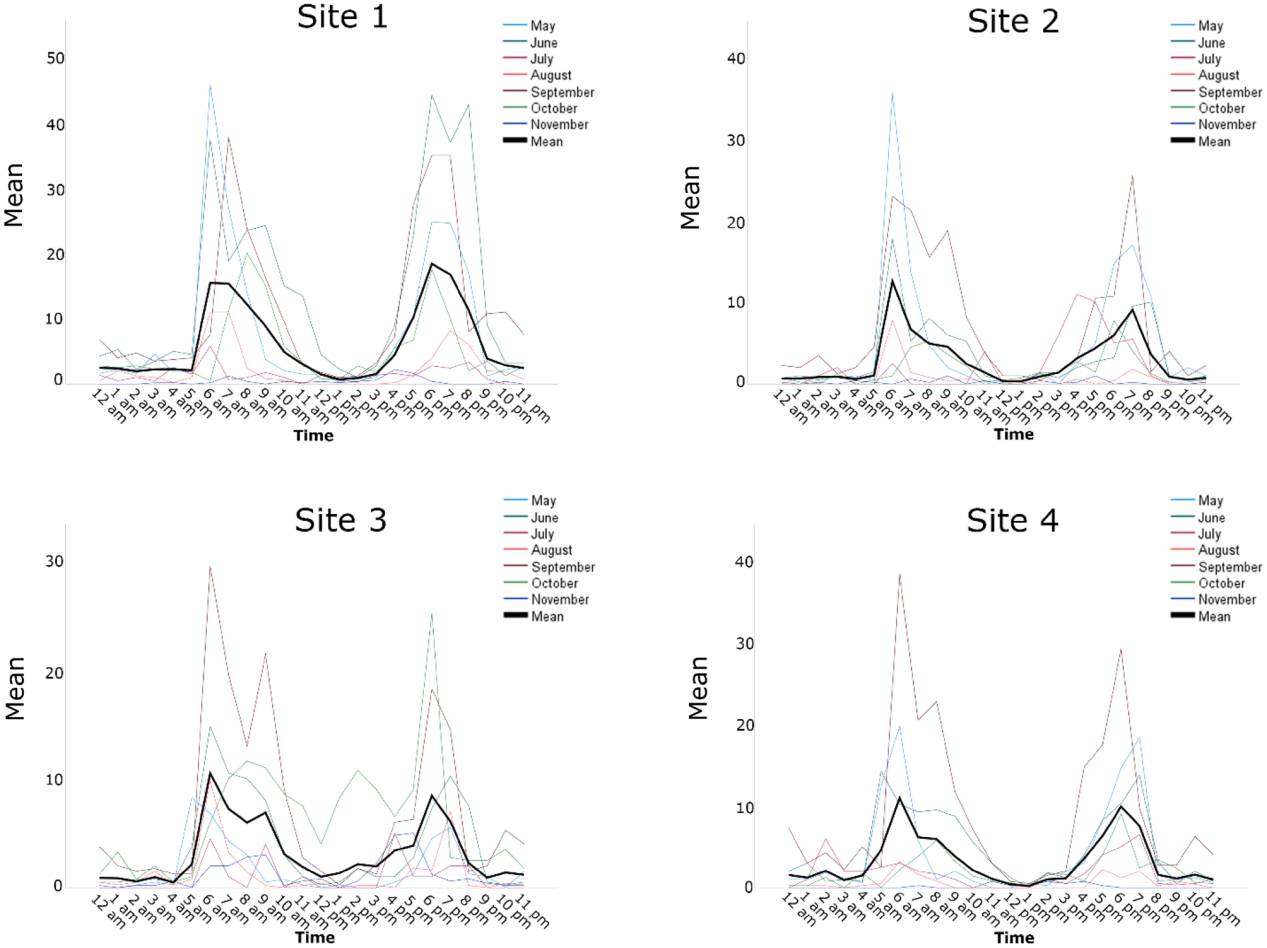
Diel activity patterns for *Ae. aegypti* populations at each of the 4 sampling sites in Brownsville, Texas. “Mean” on the graphs represent the mean number of female *Ae. aegypti* mosquitoes captured per trap/hour. The thick black line is the mean number of *Ae. aegypti* female mosquitoes captured per trap/hour throughout the study period from May to November 2019.

**Figure 3:**
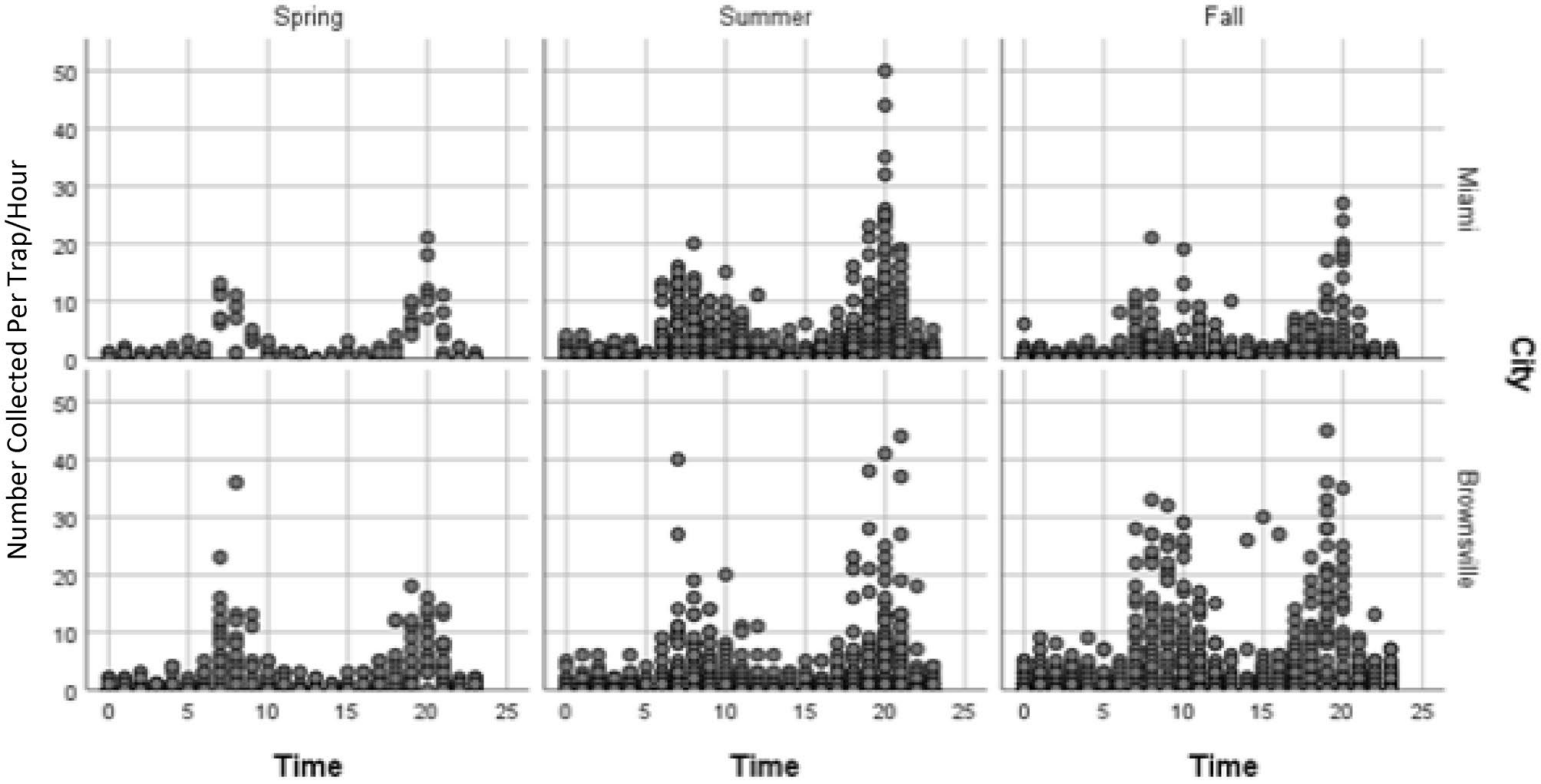
Diel activity patterns of *Ae. aegypti* in different seasons (Spring March–June, Summer June – September, Fall September–December). The y axis is the relative abundance of female *Ae. aegypti*; the number of female *Ae. aegypti* captured per trap/hour at all sites in each city.

**Figure 4:**
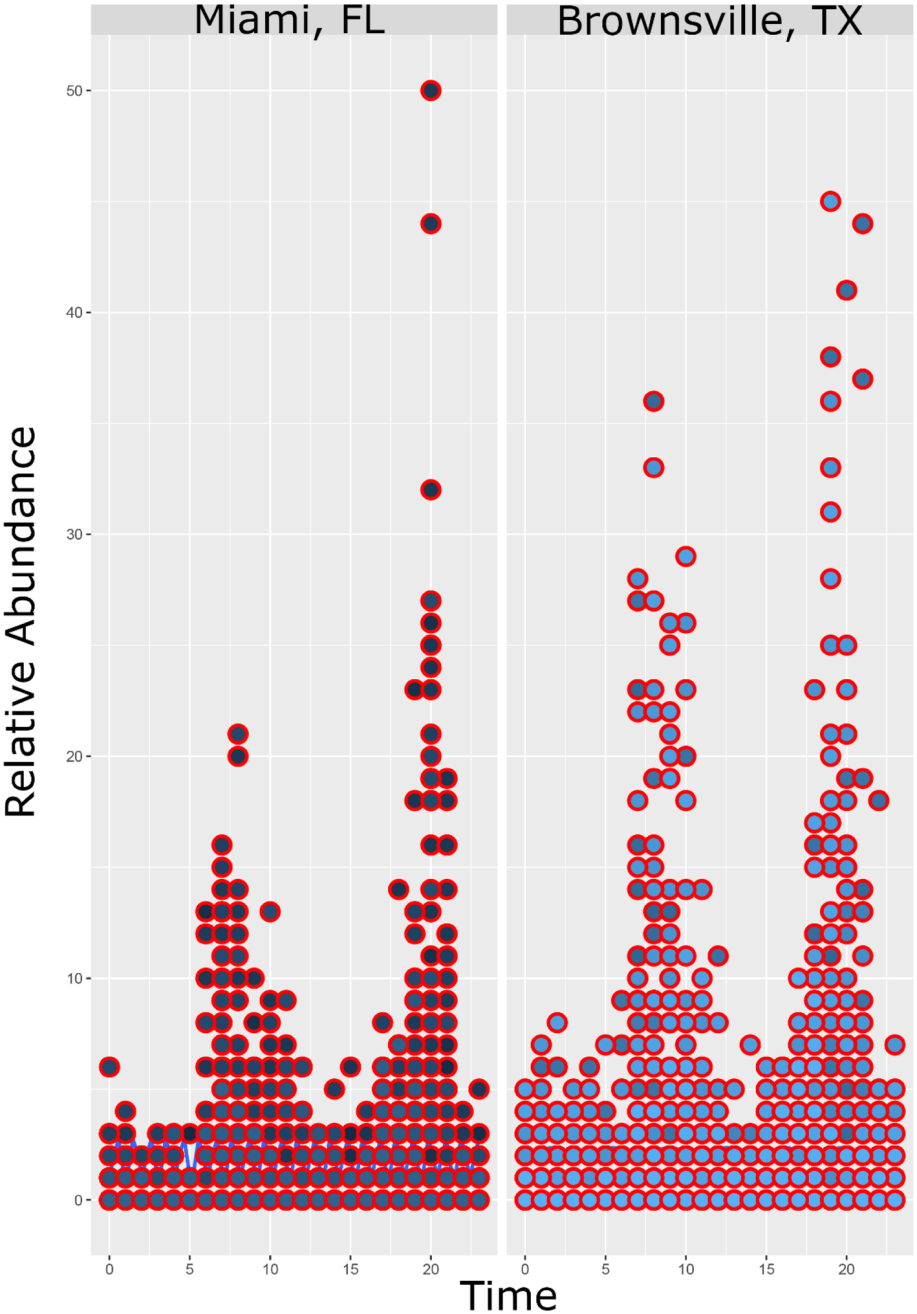
Diel activity patterns of female *Ae. aegypti* mosquitoes in Miami, Florida and Brownsville, Texas. Relative abundance on the y axis represents the relative abundance of female *Ae. aegypti* mosquitoes captured per trap per hour throughout the sampling period, May through November 2019**.**

The site-specific diel activity patterns for *Ae. aegypti* populations in Miami, FL and Brownsville, TX are summarized in Figs. 1 and 2, respectively. Data analysis showed significant differences in the *Ae. aegypti* diel activity patterns within trap locations and within seasons (Table 2, Figs. 1, 2, 3). However, these variations did not affect the overall diel activity patterns within the cities (Fig. 4). Overall, *Ae. aegypti* activity was continuous throughout the day and throughout the night with significantly elevated activity peaks in the mornings and the evenings (Figs. 1, 2, 3, 4). The morning peaks were between 7:00 and 8:00 in both Miami, FL and Brownsville, TX (Fig. 4). The evening peaks were between 19:00 and 20:00 in both Miami, FL and Brownsville, TX (Fig. 4). Multivariate analysis indicated that both morning and evening peaks were significant: Peak Start Time *P *value = 0.043, Peak Max *P *value = 0.006, suggesting consistent elevated activity at these times. In addition, the multivariate analysis indicated that the morning peak was significantly different between cities; the peak in Brownville, TX was significantly higher than that in Miami, FL (*P *value = 0.001) (Fig. 4).

**Table 2:** Results of the GLIMMIXED regression with a generalized Poisson estimator and log link to assess the effect of geographic location, seasonality, and time in the diel activity of *Ae. aegypti*.

Covariate	*F* value	*Pr* > *F*
City	1.73	0.1894
Season	5.71	**0.0038**
City(Season)	49.22	** < .0001**
Area(City*Season)	7.74	** < .0001**
Time	48.62	** < .0001**

Bold values indicate statistically significantly different.

## Discussion

Our results show that the average diel activity patterns of *Ae. aegypti* populations in both Miami, FL and in Brownsville, TX were very similar; they both had two peaks, one in the early morning and the other in the evening, and the average host-seeking peaks are between 7:00 and 8:00 and between 19:00 and 20:00 (Fig. 4). Similar observations were previously reported by several investigators^3,4,10–12^ and the bimodal diel activity pattern is the most frequently reported for *Ae. aegypti* populations worldwide. However, variations between peak activity have been detected between populations. In East Africa, for instance, Trpis et al.^3^ reported peak activity at 7:00 and at 19:00, whereas McClelland^10^ reported peak activity two or three hours after sunrise (9:00 or 10:00) and one or two hours before sunset (17:00 or 16:00). Similarly, in the United States, Smith et al.^7^ observed a bimodal diel activity pattern for *Ae. aegypti*, but the evening peak was earlier, between 17:00 and 19:00. Despite these variations, the spacing of the peaks is similar in all these studies despite the fact that these studies were conducted in ecologically and climatically diverse locations.

The activity patterns observed at site 3 in Brownsville (Fig. 2) and at site 1 in Miami (Fig. 1) were trimodal. In Brownsville, the trimodal activity peaks were between 6:30 and 7:30, 9:30 and 10:30, and 18:30 and 19:30 (Fig. 2), and in Miami the trimodal peaks were between 7:00 and 8:00, 9:00 and 10:00 and between 19:00 and 20:00 (Fig. 1). Interestingly, the timing of the third peak was similar in both Brownsville site 3 and Miami site 1 suggesting similar underlying factors despite geographic distance, different ecology, and different climate. Brownsville, Texas, is in the Lower Rio Grande Alluvial Floodplain ecoregion. The climate is humid subtropical and urbanization has removed most of the indigenous palm trees and floodplain forests vegetation (https://www.epa.gov/sites/default/files/2018-05/documents/brownsvilletx.pdf). Miami is in the Tropical Florida Ecoregion. Similar to Brownsville, Texas, urbanization and agriculture has replaced most of the indigenous Pine Rockland vegetation. Trimodal biting patterns for *Ae. aegypti* have been observed before in Trinidad by Chadee and Martinez^4^, but the middle peak was observed at 11:00 which is half an hour to an hour later than what we observed in Miami and Brownsville, respectively (Figs. 1 and 2). While the morning and evening peaks coincide with human outdoor activity, the middle peak occurs during high heat conditions and the factors that lead to this peak or its importance in the epidemiology of *Ae. aegypti*-borne arboviral diseases are currently not known. The studies by McClelland^13^ observed multiple activity peaks in an East African population of *Ae. aegypti*. The significance of the different activity patterns to the epidemiology of *Ae. aegypti*-borne arboviral diseases are currently unknown and we think they need more investigation especially since *Ae. aegypti*-borne arboviral infections have been rising in the recent past^14,15^.

We observed that the host-seeking activity peaks were consistent between 5:45 and 7:30 and between 18:00 and 20:45 (Figs. 1 and 2). These observations are important in planning and conducting control operations directed at the adult *Ae. aegypti* female populations. During the 2016 Zika outbreak, there was no specific information on the host-seeking activity patterns of *Ae. aegypti* in Miami Dade County and the adulticide treatment implemented as part of an integrated approach targeted the morning activity^16^. The integrated approach effectively reduced the vector population and interrupted the transmission of the Zika virus; however, it highlighted the need for site-specific information on the diel activity patterns of *Ae. aegypti* in Miami Dade County in particular and the CONUS in general. There have been sporadic *Ae. aegypti*-borne arboviral disease outbreaks in Miami Dade County, FL and the city of Brownsville, TX^17–21^, in the future we will be better prepared to conduct effective adulticide applications with the current knowledge of the diel activity patterns of *Ae. aegypti* in these areas. Furthermore, we are now better equipped to educate the public on how to minimize exposure to *Ae. aegypti*-borne arboviral diseases by avoiding outdoor activities during peak biting activity periods.

In our studies, we used BG-Sentinel 2 traps and monitored them every hour, twenty-four hours a day over 96 h, a method with some similarities to that used by Smith et al.^7^. In the past, diel biting activity studies were carried out using human landing catches following the methods primarily established by Haddow^22^. To our knowledge, only two studies have previously used sampling procedures not based on human landing catches to study the biting activity patterns of *Ae. aegypti*; the study by Ortega-Lopez et al.^6^ used mosquito electrocuting traps, and the study by Smith et al.^7^ used a mechanical rotator mosquito trap. In the present study, the use of BG-Sentinel II traps had the advantage that it was specifically designed to capture female host-seeking *Ae. aegypti*^8,9^. In addition, attached BG-Counter devices can keep track of the number of mosquitoes captured per specified unit time and environmental conditions, and store the information in a cloud server. However, the BG-Sentinel 2 traps collected a wide variety of mosquito species, (Table 1), and to keep track of specific species captured each hour, we had to monitor them every hour.

Overall, we present data on the diel activity of *Ae. aegypti* populations in two cities in the southern United States. In both cities the activity patterns were bimodal; there were peaks of activity in the mornings and the evenings. The significance of these observations is that these peaks can be targeted to improve the effectiveness of adulticide treatments aimed at controlling *Ae. aegypti* adult populations. Using BG-Sentinel 2 traps eliminates individual variations associated with human landing catches and the associated danger of infections from wild mosquitoes especially during ongoing outbreaks.

## Methods

### Study Sites

**Figure 5:**
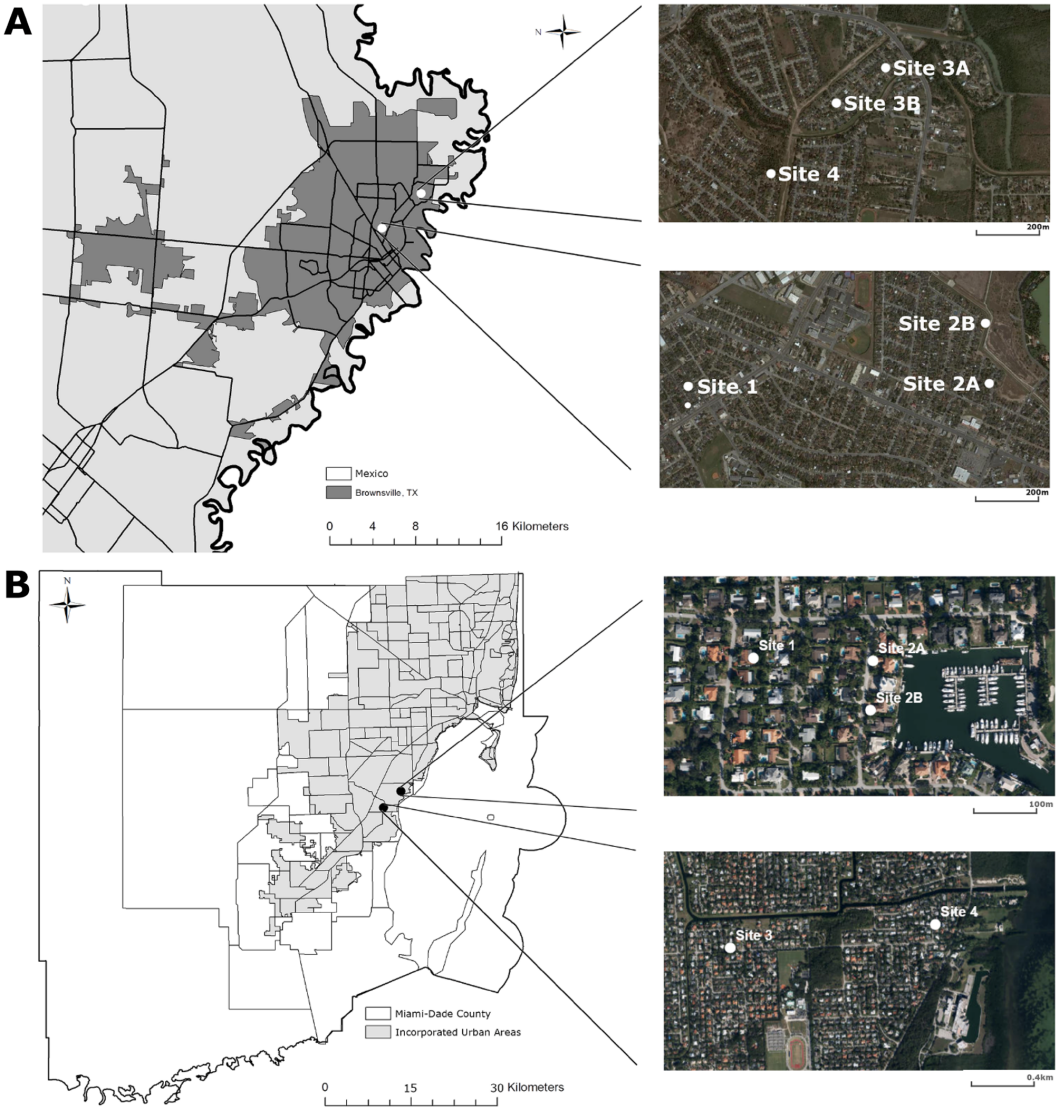
Maps of sampling sites in Brownsville, Texas (**A**) and Miami Dade County, Florida, (**B**). In Brownsville, the traps were moved from 2A to 2B and from 3A to 3B because mosquito caught dipped markedly at sites 2A and 3A. The traps were moved to the more productive sites 2B and 3B. In Miami, the trap was moved from site 2A to site 2B for the same reason as in Brownsville. The figure was produced using ArcGIS 10.2 (Esri, Redlands, CA), using freely available layers from the Miami-Dade County’s Open Data Hub—https://gis-mdc.opendata.arcgis.com/.

Miami, FL (25.7617° N, 80.1918° W) and Brownsville, Texas (25.9017° N, 97.4975° W) were selected as study sites; these cities have a history of *Ae. aegypti* arboviral outbreaks^17–21^. Four study sites were selected in each city (Fig. 5). All sites were in front or back yards in private homes and selected after obtaining permission from the owners.

### Sample collection

We used BG-Sentinel-2 traps (Biogents AG, Regensburg, Germany) equipped with BG Counters (Biogents AG, Regensburg, Germany). The traps were baited with BG Lures and dry ice as a source of carbon dioxide^23,24^. These traps were used as substitutes for human hosts; they use lures which are blends of mosquito attractants consisting of lactic acid, ammonia, and caproic acid, substances which are also found on human skin^8^. The lures imitate human odor and the carbon dioxide imitates human breath. Owing to the lack of automatic collecting devices for BG-Sentinel traps, the traps were physically monitored every hour for 96 h (4 days) at each location once every month from May to November 2019. Because of workforce limitations we did not have field teams dedicated to specific traps, rather a single team visited all four traps to collect the mosquitoes captured and reset each trap once every hour. Trap 1 (site 1) was monitored at the top of the hour, trap 2 (site 2) at 15 min past the hour, trap 3 (site 3) at 30 min past the hour and trap 4 (site 4) at 45 min past the hour. To accomplish this the field crews worked either in 12-h shifts (two teams) or eight-hour shifts (three teams). On each visit, the BG-Sentinel collection bag was removed from the trap and replaced with a fresh one. The removed bag was placed into a gallon ziplock bag labeled with the trap site and the collection time and taken to the lab on ice in a cooler. In the lab, the mosquitoes were identified to species on chill tables by using the keys of Darsie and Ward^25^, placed in labeled cryovials, and shipped on dry ice to the CDC lab in Fort Collins, CO for arbovirus testing. The species and number of mosquitoes collected were recorded on Excel spreadsheets.

In June, three traps (one in Miami and two in Brownsville) resulted in low *Ae. aegypti* catch so they were relocated to more productive sites within the same neighborhood to better evaluate diel activity patterns. Trapping was conducted from May 2019 through November 2019. On each sampling, trap data was collected hourly for 4 days (96 h). In Miami, collections were conducted from 5/7/19 to 5/11/19, 6/3/19 to 6/7/19, 7/7/19 to 7/11/19, 8/4/19 to 8/8/19, 9/3/19 to 9/7/19, 10/6/19 to 10/10/19, and 11/11/19 to 11/14/19. In Brownsville, collections were conducted 5/20/19 to 5/24/19, 6/13/19 to 6/16/19, 7/21/19 to 7/25/19, 8/18/19 to 8/22/19, 9/15/19 to 9/19/19, 10/7/19 to 10/11/19 and 11/22/19 to 11/27/19.

### Data analysis

The data within trap locations were considered the unit identifier in a Multi-level Multi-variable Longitudinal Model. The repeated measure was time in hours within each trap location*date. Then, we used the GLIMMIXED in SAS 9.4 and transformed the data as log(*Aedes aegypti* + 1) with a generalized Poisson estimator and log link with only linear time and no random intercept as the random effect. The covariates Linear Time*City*Season and Linear Time* Trap Location*City*Season were not significant and were removed from the model. We then used a multivariate analysis to analyze peak differences in the data, days with no peaks were removed and the morning peak (before 1500 h) and evening peak (after 1500 h) were analyzed separately using the Peak Start Time and Peak Max using the peak detection and measurement spreadsheet (available at: https://terpconnect.umd.edu/~toh/spectrum/PeakFindingandMeasurement.htm).

## References

[1] Christophers SR (1960). Aedes aegypti (L) the Yellow Fever Mosquito: Life History.

[2] Lumsden WHR (1957). The activity cycle of domestic *Aedes* (*Stegomyia*) *aegypti* (L.) (Diptera, Culicidae) in Southern Province, Tanganyika. Bull. Ent. Res..

[3] Trpis M, McClelland GAH, Gillett JD, Teesdale C, Rao TR (1973). Diel periodicity in the landing of *Aedes aegypti* on man. Bull. World Health Org..

[4] Chadee DD, Martinez R (2000). Landing periodicity of *Aedes aegypti* with implications for dengue transmission in Trinidad, West Indies. J. Vector Ecol..

[5] Diarrassouba S, Dossou-Yovo J (1997). Atypical activity rhythm in *Aedes aegypti* in a sub-Sudanian savannah zone of Côte d'Ivoire. Bull. Soc. Pathol. Exot..

[6] Ortega-López LD, Pondeville E, Kohl A, León R, Betancourth MP, Almire F, Sergio T-V, Saldarriaga S, Mirzai N, Ferguson HM (2020). The mosquito electrocuting trap as an exposure-free method for measuring human-biting rates by *Aedes* mosquito vectors. Parasit. Vectors.

[7] Smith M, Dixon D, Bibbs C, Autry D, Xue RD (2018). Diel patterns of *Aedes aegypti* (*Diptera*: *Culicidae*) after resurgence in St. Augustine, Florida as collected by a mechanical rotator trap. J. Vector Ecol..

[8] Krockel U, Rose A, Eiras AE, Geier M (2006). New tools for surveillance of adult yellow fever mosquitoes: comparison of trap catches with human landing rates in an urban environment. J. Am. Mosq. Control Assoc..

[9] Maciel-de-Freitas R, Eiras AE, Lourenço-de-Oliveira R (2006). Field evaluation of effectiveness of the BG-Sentinel, a new trap for capturing adult *Aedes aegypti* (*Diptera*: *Culicidae*). Mem. Inst. Oswaldo Cruz.

[10] McClelland GAH (1959). Observations on the mosquito, *Aedes* (*Stegomyia*) *aegypti* (L.) in East Africa I. The biting cycle in an outdoor population at Entebbe, Uganda. Bull. Entomol. Res..

[11] Boorman MA (1960). Studies on the biting habits of the Mosquito *Aedes* (*Stegomyia*) *aegypti* Lin, in a West Africa village. W. Afr. Med. J..

[12] Diouf G, Seck MT, Ciss M, Faye B, Biteye B, Bakhoum MT, Fall AG (2021). Improving the efficiency of the BG sentinel 2 trap to assess the activity of *Aedes* (*Stegomyia*) *aegypti* [Linnaeus, 1762] in Senegal. Acta Trop..

[13] McClelland GAH (1960). Observations on the mosquito *Aedes* (*Stegomyia*) *aegypti* (L.) in East Africa II. The biting cycle in a domestic population on the Kenya Coast. Bull. Entomol. Res..

[14] Rogers DJ, Wilson AJ, Hay SI, Graham AJ (2006). The global distribution of yellow fever and dengue. Adv. Parasitol..

[15] Bhatt S, Gething PW, Brady OJ, Messina JP, Farlow AW, Moyes CL, Drake JM, Brownstein JS, Hoen AG, Sankoh O, Myers MF, George DB, Jaenisch T, Wint GR, Simmons CP, Scott TW, Farrar JJ, Hay SI (2013). The global distribution and burden of dengue. Nature.

[16] McAllister JC, Porcelli M, Medina JM, Delorey MJ, Connelly CR, Godsey MS, Panella NA, Dzuris N, Boegler KA, Kenney JL, Kothera L, Vizcaino L, Lenhart AE, Mutebi JP, Vasquez C (2016). Mosquito control activities during local transmission of Zika virus, Miami-Dade County, Florida, USA. Emerg. Infect. Dis..

[17] Ramos MM, Mohammed H, Zielinski-Gutierrez E, Hayden MH, Lopez JLR, Fournier M, Trujillo AR, Burton R, Brunkard JM, Anaya-Lopez L (2008). Epidemic dengue and dengue hemorrhagic fever at the Texas-Mexico border: results of a household-based seroepidemiologic survey, December 2005. Am. J. Trop. Med. Hyg..

[18] Likos A, Griffin I, Bingham AM, Stanek D, Fischer M, White S, Hamilton J, Eisenstein L, Atrubin D, Mulay P, Scott B, Jenkins P, Fernandez D, Rico E, Gillis L, Jean R, Cone M, Blackmore C, McAllister J, Vasquez C, Rivera L, Philip C (2016). Local mosquito-borne transmission of Zika virus - Miami-Dade and Broward Counties, Florida, June-August 2016. Morb. Mortal. Wkly. Rep..

[19] Duffy J (1968). Yellow fever in the continental united states during the nineteenth century. Bull NY Acad. Med..

[20] Adalja AA, Sell TK, Bouri N, Franco C (2012). Lessons learned during dengue outbreaks in the United States, 2001–2011. Emerg Infect Dis..

[21] Bouri N, Sell TK, Franco C, Adalja AA, Henderson DA, Hynes NA (2012). Return of epidemic dengue in the United States: implications for the public health practitioner. Public Health Rep..

[22] Haddow AJ (1954). Studies of the biting habits of African mosquitoes. An appraisal of methods employed, with special references to the twenty-four hour catch. Bull. Entomol. Res. Bull..

[23] Ázara TMFd, Degener CM, Roque RA, Ohly JJ, Geier M, Eiras AE (2013). The impact of CO_2_ on collection of *Aedes aegypti* (Linnaeus) and *Culex quinquefasciatus* Say by BG-Sentinel (R) traps in Manaus Brazil. Mem. Inst. Oswaldo Cruz.

[24] Wilke ABB, Carvajal A, Medina J, Anderson M, Nieves VJ, Ramirez M, Vasquez C, Petrie W, Cardenas G, Beier JC (2019). Assessment of the effectiveness of BG-Sentinel traps baited with CO_2_ and BG-Lure for the surveillance of vector mosquitoes in Miami-Dade County, Florida. PLoS ONE.

[25] Darsie RF, Ward RA (2005). Identification and Geographic Distribution of the Mosquitoes of North America, North of Mexico.

